# The Particularities of Arterial Hypertension in Female Sex: From Pathophysiology to Therapeutic Management

**DOI:** 10.3390/jcm14093137

**Published:** 2025-05-01

**Authors:** Antonios Lazaridis, Anastasia Malliora, Eugenia Gkaliagkousi

**Affiliations:** 1 1st Department of Internal Medicine, Papageorgiou General Hospital, 56429 Thessaloniki, Greece; spanbiol@hotmail.com; 2 3rd Department of Internal Medicine, Papageorgiou General Hospital, Aristotle University of Thessaloniki, 56403 Thessaloniki, Greece; anamalliora@yahoo.gr

**Keywords:** hypertension, women, hormone, cardiovascular risk, antihypertensive treatment

## Abstract

Arterial hypertension is the most important modifiable cardiovascular risk factor and a major cause of cardiovascular mortality worldwide. In daily clinical practice, the hypertensive patient is often treated in a uniform way, thus ignoring the significant effects of sex on several aspects of hypertension, including its prevalence, pathophysiology, response to antihypertensive treatment, and outcomes. Along with the immune response and several cardiometabolic risk factors that frequently coexist, the substantial hormonal changes during a woman’s life cycle are among the main pathophysiological mechanisms driving hypertension in women. Concurrently, women exhibit increased cardiovascular risk at lower blood pressure (BP) levels compared to age-matched men and present certain disparities in the incidence of cardiovascular events and subsequent hypertension-related cardiovascular prognosis. In addition, women respond differently to antihypertensive treatment, experience more drug-related side effects, and exhibit lower rates of BP control compared to men. Currently, international guidelines propose the same targets and the same therapeutic algorithms for the treatment of hypertension in both sexes without taking into account the sex differences that exist. In this review, we aim to describe certain particularities of arterial hypertension in the female sex, moving from pathophysiological aspects to clinical and therapeutical management.

## 1. Introduction

Arterial hypertension is the most important modifiable risk factor for cardiovascular disease worldwide and a major cause of cardiovascular mortality and disability in both sexes [1]. Notably, in recent years, a growing amount of evidence has highlighted the impact of sex on several aspects of hypertension, including its pathophysiology, prevalence, cardiovascular prognosis, and antihypertensive treatment response. In fact, multiple sex-related differences exist that may arise as early as the beginning of a woman’s reproductive age [2,3,4].

The constant change in the hormonal milieu throughout a woman’s cycle represents a notable factor implicated in the pathophysiology of hypertension in females. Typically, several conditions that arise from that hormonal imbalance confer a significantly higher risk of developing hypertension in women compared to men, the most impactful of them being pregnancy and menopause [5]. Furthermore, various other factors that are influenced by sex contribute to hypertension in females, such as the upcoming role of the immune system and certain cardiometabolic risk factors that cluster with higher frequency in women, including metabolic syndrome, obesity, insulin resistance, and chronic autoimmune inflammatory diseases [4].

In addition to the pronounced pathophysiological differences that exist between the two sexes, women respond differently to cardiovascular risk in association with blood pressure (BP). In this regard, it has been demonstrated that the risk of major cardiovascular events occurs at lower BP levels in females compared to males [6]. Moreover, the impact of hypertension on the incidence of cardiovascular events seems to differentiate according to sex, with women suffering more frequently from heart failure and myocardial infarction [7,8].

Finally, women differ in several aspects of the antihypertensive treatment compared to men. More specifically, it has been shown that women differ in terms of drug metabolism and are also more prone to experiencing certain drug-related side effects compared to men [9,10]. At the same time, women seem to present comparable BP reductions with men upon initiating all major antihypertensive drug classes, but noticeably, they present poorer BP control [11,12].

Despite all the above fundamental differences, current international guidelines do not support a sex-specific approach for hypertensive patients, including pre- and postmenopausal women. In this review, we aim to describe the particularities of arterial hypertension in the female sex, from pathophysiological aspects to its therapeutic management.

## 2. Epidemiology of Hypertension in Women

In general, the prevalence of hypertension is higher in men <50 years old compared to age-matched premenopausal women. From puberty onwards, however, certain sex differences in the BP trajectories over the life course exist, with the mean BP being approximately 10 mmHg higher in male subjects by the age of 18 [13]. This pattern begins to reverse by the third decade of life, where BP exhibits a steeper incline in women. Of note, the prevalence of hypertension in women increases significantly after the age of 60 [14]. As a result, the prevalence of hypertension reaches almost 68% in postmenopausal women aged 65–74 years, exceeding the prevalence of men of the same age [14,15]. Remarkably, the prevalence of hypertension in women aged ≥75 years reaches 78% [16].

## 3. Pathophysiology of Hypertension in Women

### 3.1. The Role of Sex Hormones

**Figure 1:**
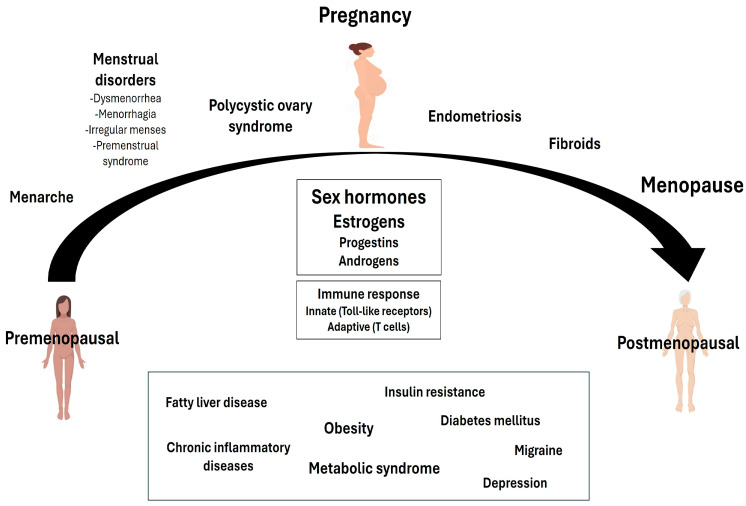
Factors contributing to the pathophysiology of hypertension in women.

The pathophysiology of hypertension in women is elaborate and multifactorial and includes a variety of sex-related factors [4,17] (Figure 1), such as the female sex hormones and their age-related changes due to declining ovarian function. It has long been established that estrogens exert several potent vasoprotective effects, including (i) increased nitric oxide (NO) bioavailability [18,19] and stimulation of adenosine and prostacyclin synthesis [20], (ii) interception of the vascular response to injury through decreased expression of adhesion molecules, inhibition of neointima formation and suppression of the mitogenic effects of several factors generated at the sites of endothelial injury [21], (iii) decrease in basal sympathetic nervous system activity [22,23], and, most importantly, (iv) suppression of the renin–angiotensin–aldosterone system (RAAS) [18,24,25]. At the same time, estrogen depletion reverses the above beneficial effects, thus conferring a pro-hypertensive potential. In this context, studies in estrogen-deficient postmenopausal women have shown alterations in the autonomic nervous system, including decreased baroreflex sensitivity and vagal tone and a preponderance of the sympathetic tone [26]. In addition, estrogen depletion has been linked to enhanced angiotensin II (AngII) activity, leading to impaired renal sodium handling and oxidative stress [27,28]. Consistent with the above, it has been demonstrated that salt-loading induces a decrease in renal plasma flow in postmenopausal women, and surgical menopause has been linked to salt-sensitive hypertension [29,30].

Although a wealth of evidence highlights the implication of estrogens in the pathophysiology of hypertension, the role of progestins and androgens should also be taken into account. In this context, experimental studies have shown that progesterone, the natural progestin, exerts an endothelium-independent vasodilatory effect [31]. Additionally, clinical data have shown that administration of progesterone in combination with estrogens produces a greater reduction in systolic BP (SBP) than estrogens alone in postmenopausal women [32]. On the other hand, the role of androgens in the pathophysiology of hypertension in women remains controversial and under-investigated. Experimental data have shown that androgens exert a rather pro-hypertensive effect [33,34,35]. From a clinical point of view, women who have hyperandrogenemia, such as those with polycystic ovary syndrome (PCOS), present hypertension, oxidative stress, and an increased inflammatory milieu [36].

### 3.2. Hormonal-Related Conditions Associated with Hypertension: From Physiology to Clinical Disease

Considering the mechanistic involvement of sex hormones in vascular homeostasis, many physiological and pathological conditions affecting the menstrual cycle during a woman’s lifetime have been associated with hypertension, even though the concurrent influence of other major extrinsic factors such as obesity, smoking, salt consumption, and sedentary lifestyle must be taken into account [13].

#### 3.2.1. Menarche

Menarche, or first menstruation, represents the start of the menstrual life cycle of a woman, which typically occurs between the ages of 10 and 16 [37]. Of note, menarche age has been correlated with increased cardiovascular risk later in adulthood, including hypertension [38,39]. Relative to this, a U-shaped association has been observed between age at menarche and hypertension, linking early (≤12 years) or late menarche (≥16 years) with the highest risk of hypertension [40,41,42]. Interestingly, the risk of hypertension has been found to be substantially increased in women with additional key risk factors, including high body mass index (BMI), psychological stress, passive smoking, and an imbalanced diet [41].

#### 3.2.2. Menstrual Disorders

Disorders of the menstrual cycle consist of premenstrual syndrome, irregular, painful (dysmenorrhea), and heavy (menorrhagia) menstruation. In a retrospective study of 704,743 female individuals aged 18–40 years who were followed up for 26 years, women with irregular menstrual cycles presented elevated hypertension risk compared to those with regular menses [43]. Above all, premenstrual syndrome, which is a very disruptive menstrual irregularity, has been associated with certain alterations in the female vascular physiology that could predispose to hypertension [44], such as increased carotid-to-femoral pulse wave velocity (PWV) and pulse pressure (PP) [45]. From a clinical perspective, a prospective study by Bertone-Johnson E. et al. found that women with moderate to severe premenstrual syndrome had a 40% higher risk of developing hypertension over a follow-up period of 20 years compared with asymptomatic women. Notably, the observed risk did not vary by BMI and concurrent medications and was highest among women younger than 40 years [46].

#### 3.2.3. Pregnancy

Many physiological changes that occur during pregnancy in order to meet the increased maternal and fetal demands are held responsible for the development of gestational hypertension, including changes in cardiac output and kidney function, mediated by increased RAAS activation and subsequent salt and water retention. Additionally, progressive insulin resistance and weight gain also result in enhanced sympathetic activity, endothelial dysfunction, and increased vascular resistance, all factors predisposing to hypertension [5,47,48]. Importantly, even though gestational hypertension may resolve within 6 weeks postpartum, it may also adversely impact maternal cardiovascular health later in life. According to this, it is now well documented that women with a history of gestational hypertension present an increased risk of developing hypertension late in life, which may be evident as early as the first years following birth [49,50,51,52]. As such, a recent study showed that women with gestational hypertension at their first pregnancy exhibit an almost three-fold higher risk of developing hypertension compared to women with normotensive pregnancies. In particular, the risk was higher within the first five years postpartum, reaching a 4.3-fold increase rate [53]. Another cohort of more than one million women showed an exceptionally high rate of incident hypertension (up to 25-fold) within one year postpartum in women with gestational hypertension compared to women with a normotensive pregnancy. Interestingly, the risk was significantly elevated across a period of 20 years postpartum [54]. Moreover, another study adjusting for a wide range of pre-pregnancy risk factors found a persistent seven-fold excess risk of hypertension within 10 years of delivery in women with a history of gestational hypertension [55].

Finally, preeclampsia, which is the most severe phenotype among the hypertensive disorders of pregnancy, is equally linked to a substantial risk of developing hypertension. A wealth of studies has confirmed that a history of preeclampsia during pregnancy is associated with a two-fold to seven-fold higher risk of developing hypertension over 10 years postpartum [51,54,55,56,57,58]. In addition to this, a history of preeclampsia is associated, in the long term, with a substantially increased burden of cardiovascular risk factors all associated with hypertension, including higher serum uric acid and urinary albumin levels, carotid atherosclerosis, and impaired endothelial function [59,60].

#### 3.2.4. Menopause

Menopause marks a major hormonal change during a woman’s life. It is characterized by a dramatic decrease in estrogen levels, which has a significant impact on several physiologic pathways, ultimately leading to endothelial dysfunction, oxidative stress, impaired sodium excretion, increased arterial stiffness, and enhanced sympathetic nervous activity [61,62,63,64,65]. Typically, the prevalence of hypertension reaches its highest rate in postmenopausal women and is twice as high compared to premenopausal females [66]. Although menopause has been considered as a contributing mechanism to the development of postmenopausal hypertension, it is currently controversial whether endogenous estrogen depletion accounts, independently, for the higher prevalence of hypertension in postmenopausal women. In fact, several data from cross-sectional studies have demonstrated an increase [67], a neutral effect [68], or even a decrease in BP values [69] with the onset of menopause. Furthermore, prospective data have shown that menopause is not associated with BP increase [70], while other prospective studies have demonstrated that the onset of menopause may independently increase BP, primarily SBP [71]. The overall controversy is due to the fact that aging, a major risk factor of hypertension, as well as increasing BMI coincide with postmenopausal status and, thus, take their share as crucial determinants of hypertension, confounding the observed results [62,72,73,74]. Moreover, several other study-related factors confound the results, such as sample size, age ranges, duration of postmenopausal status, self-reported menopause based on questionnaires, and, finally, the inclusion of women with surgical menopause.

#### 3.2.5. PCOS

PCOS is the most common endocrine disease affecting up to one in five women of reproductive age. Many adverse cardiometabolic conditions cluster with increased prevalence in PCOS, including obesity, hyperglycemia, dyslipidemia, metabolic-associated fatty liver disease, and hypertension [75]. Subsequently, several mechanisms have been postulated to explain the association between hypertension and PCOS, including activation of the RAAS and sympathetic nervous system, hyperinsulinemia, hyperandrogenism, and endothelial dysfunction [76,77].

So far, several studies have shown an increased prevalence of hypertension in women with PCOS compared to the general population [76,78]. However, they are complicated by a failure to adjust for the presence of obesity, which is highly prevalent in women with PCOS and, at the same time, a well-established risk factor of hypertension. On the other hand, a handful of studies have included BMI in their analyses, and even though results are conflicting, a particular association between PCOS and the risk of hypertension cannot be precluded [76,79,80,81,82,83]. Towards this direction, the Australian Longitudinal Study on Women’s Health, including 8223 women of childbearing potential (mean age 25 years), showed that PCOS was independently associated with a 37% greater risk of incident hypertension over a follow-up period of 15 years. Following stratification by BMI, the incidence of hypertension was nearly four-fold higher in the obese (BMI ≥ 30 kg/m^2^) compared to the age-matched lean women [83]. Additionally, a recent meta-analysis confirmed a greater risk of hypertension of up to 1.7-fold in women with PCOS compared to age-matched controls. However, that risk was observed only in women of reproductive age and not in menopausal individuals who had suffered from PCOS during their reproductive years, thus downgrading the importance of PCOS as a predisposing risk factor for hypertension later in life [80].

#### 3.2.6. Endometriosis

Endometriosis, which affects up to 10% of women of childbearing potential, has been linked to an elevated risk of developing hypertension, probably through systemic inflammatory-mediated pathways, even though other mechanisms such as chronic nonsteroid anti-inflammatory drugs use may be involved [84]. A large prospective study including 116,430 females aged 25–42 years who were followed up for 20 years showed that endometriosis is associated with a 14% higher risk of developing hypertension compared to controls. In particular, the risk was higher in women younger than 40 years but decreased with advanced age [85]. In addition, it has been found that endometriosis is an independent risk factor for gestational hypertension and preeclampsia [86,87,88].

#### 3.2.7. Fibroids

Uterine fibroids are reported in up to 30% of women of reproductive age, and even though they are frequently overlooked, an increasing amount of evidence has linked fibroids to a notably worse cardiovascular risk profile, including hypertension [89]. Whether this association is explained by enhanced smooth muscle proliferation, hypercontractility of resistance arteries, or urinary tract obstruction by the expanding uterine masses remains to be elucidated. Either way, a cross-sectional study including 5552 women aged 20–54 years showed a significantly higher prevalence of hypertension in women with fibroids compared to controls (33.4% vs. 15.3%, respectively), the risk being higher in the younger counterparts (≤35 years) [90]. Moreover, higher odds of hypertension-mediated asymptomatic organ damage, primarily driven by increased PP and aortic PWV, have been reported in young women (<50 years) with fibroids compared to controls (66.7% vs. 42.9%, respectively), even though the association is attenuated after adjustment for age and BMI [91]. Finally, fibroids have been associated with a higher risk of developing a hypertensive disorder during pregnancy [92].

##### The Role of the Immune System

Over the last few years, growing data have highlighted the role of immunity in the pathophysiology of hypertension. However, much remains unknown regarding the effect of sex on the immune-driven environment of hypertension and the development of vascular damage. Contemporary studies in experimental hypertension have confirmed the presence of distinct sex differences considering the adaptive immune response, mainly within the T-cell niche [93,94,95,96]. In this context, it has been shown that premenopausal female animals have a predilection for anti-inflammatory T regulatory cells and the production of anti-inflammatory cytokines, such as interleukin-10, which confer a largely protective effect from hypertension. At the same time, menopause abrogates this protective effect, which may be due to changes in the population of anti-inflammatory T regulatory cells [94,97,98]. On the other hand, scarce experimental data regarding sex differences in innate immune cells exist. Only recently have the sex differences in response to Toll-like receptor (TLR) 3,4,9 signaling emerged [99,100].

##### The Role of Cardiometabolic and Other Risk Factors

Various cardiometabolic risk factors that cluster throughout a woman’s life cycle also contribute to the pathophysiology of hypertension. Women, especially postmenopausal, are more prone to develop metabolic syndrome and obesity [101,102]. Furthermore, changes in weight and body fat distribution are linked to insulin resistance, diabetes mellitus (DM), fatty liver disease, and hypertension [103]. In addition, women, compared to men, show a higher prevalence of certain diseases that are closely related to the development of hypertension and cardiovascular disease, such as chronic inflammatory diseases (rheumatoid arthritis, systemic lupus erythematosus) and migraine [66,104].

## 4. Clinical Implications

### 4.1. BP Levels Associated with Cardiovascular Risk in Women

It has been consistently demonstrated that the risk of CVD continuously increases from the SBP level of 120 mmHg and upwards. However, studies have advocated that the association of SBP with incident CVD may be influenced by sex. As such, in women, it has been supported that the risk of major cardiovascular events such as stroke, myocardial infarction, and heart failure occurs at lower BP levels, including SBP values approximately 10 mmHg lower than the corresponding values in men [6,105]. Indeed, a pooled analysis of 27,542 participants from established community-based cohort studies (Framingham Heart Study, Multi-Ethnic Study of Atherosclerosis, Atherosclerosis Risk in Communities Study, Coronary Artery Risk Development in Young Adults Study) showed that SBP levels of 100–109 mmHg relative to levels of SBP <100 mmHg were consistently associated with incident CVD in women but not men. In addition, the magnitude of risk seen in men at higher SBP thresholds was comparable to that seen in women at lower SBP thresholds [6].

### 4.2. Hypertension-Related Cardiovascular Prognosis in Women

Of great importance, not only cardiovascular events present at lower BP thresholds in women but also the impact of hypertension on the incidence of cardiovascular events seems to differentiate according to sex. Indeed, data from the prospective REGARDS study in 26,461 individuals aged ≥45 years showed that the association between increasing hypertension severity and incident ischemic stroke was almost twice as large in women compared with men, even after adjustment for other conventional stroke risk factors. In fact, when SBP was treated as a continuous variable, women had a higher risk of stroke compared to men per each 10 mmHg increase in SBP [106]. Furthermore, it has been speculated that the association of hypertension with cognitive decline is stronger in women compared to men. In support of this, a population-based cohort including young, middle-aged, and older adults spanning 20–76 years showed that incident midlife hypertension is associated with greater memory decline in late life in women compared to men [107]. Similarly, another population-based cohort demonstrated that hypertension during mid-adulthood (overall mean age 44 years) was associated with a 65% higher risk of dementia in women but not men. Interestingly, within the female group, hypertensive women with onset of hypertension during mid-adulthood presented 73% higher dementia risk compared to those who remained normotensive throughout their whole age span. On the contrary, there was no evidence that hypertension or changes in hypertension increased dementia risk among men [108].

Concerning the cardiovascular system, women, compared to men, typically present a greater prevalence of left ventricular (LV) hypertrophy and heart failure, outweighing men by around 2:1 in terms of the prevalence of heart failure with preserved ejection fraction (HFpEF) [8,109,110]. Notably, hypertension is associated with a three-fold higher risk of heart failure in women compared to a two-fold risk in men [111,112]. The pathophysiological role of hypertension in the development of heart failure is well established and is primarily related to chronic LV pressure overload leading to diastolic dysfunction, reduced relaxation, and increased filling pressures. The above pathophysiology is particularly impacted in older women where accelerated vascular aging, characterized by increased peripheral vascular resistance, steepened aortic PWV increase, and elevated augmentation index, further increases the cumulative pulsatile load exposure [113,114]. In addition, it has been observed that increased vascular stiffness in elderly females is closely linked to an age-related increase in LV systolic stiffness, therefore creating an altered ventricular–arterial coupling mechanism, which adversely impacts cardiovascular reserve function [115,116]. Overall, the above-described phenomena largely explain the predominance of the HFpEF phenotype in females.

Moreover, a close association between hypertension and myocardial infarction in women exists. In the UK Biobank study, which included 471,998 people (56% female) aged 40–69 years who were free of CVD at baseline, the relative risk of myocardial infarction in hypertensive women was 83% higher compared to men and consistently higher across different hypertension stages, even though the absolute risk and incidence of myocardial infarction were clearly higher in males [7]. Noteworthy, the risk in hypertensive women remained more elevated even in those receiving antihypertensive treatment as compared to treated hypertensive men. In keeping with these findings, another study including 1,25 million patients and 11,029 myocardial infarction events demonstrated a higher relative risk of myocardial infarction with increasing SBP in women compared to men [117]. Of great interest, the relationship between hypertension and cardiovascular events in females has been observed across different hypertension phenotypes. In a sex-stratified analysis in younger women aged 20–39 years with a median follow-up of 13.2 years, the hazard ratios for CVD events (myocardial infarction, stroke, heart failure, and cardiovascular disease-related death) associated with elevated BP, including isolated systolic, isolated diastolic and systolic–diastolic hypertension were higher in women compared to men, with the women-to-men relative risk ratio ranging from 1.14 to 1.46 [118]. Finally, a meta-analysis including 9357 subjects from 11 populations, of whom 47% were women, showed a steeper increase in the risk of cardiovascular events with increasing levels of 24 h and nighttime SBP in women. Consequently, for each 1-standard deviation (SD) decrease in 24-h SBP (13.4 mmHg) and nighttime SBP (14.1 mmHg), the proportion of potentially preventable events was higher in women than in men regarding all cardiovascular events (35.9% vs. 24.2% for 24 h SBP, 35.1% vs. 19.4% for nighttime SBP), therefore unveiling an extensive potential for cardiovascular prevention by lowering BP in women [119].

## 5. Therapeutic Considerations

### 5.1. Limitations in the Treatment Approach According to Sex

Even though pronounced biological differences are implicated in the pathogenesis of hypertension in women, there is still no clear sex-specific treatment approach for hypertension. As a matter of fact, both the latest European Society of Hypertension and European Society of Cardiology guidelines suggest the same ΒP goals and the same therapeutic algorithms for the management of hypertension in males and females [15,120]. More importantly, up to now, limited data and several areas of uncertainty exist regarding the BP thresholds for initiation of antihypertensive treatment in women, the therapeutic goals, the choice of antihypertensive drugs and their effectiveness, and finally, the drug-related adverse effects. This is partly related to the fact that women, including those belonging to special categories (e.g., pregnant women, pre- and postmenopausal women), are largely underrepresented in large clinical studies, including approximately 30% of the participants, and their cardiovascular risk is often underestimated [121]. Moreover, no randomized controlled trials with the necessary power to investigate BP outcomes and mortality exclusively in hypertensive women exist. Another drawback of several studies is the lack of performance of risk stratification by sex. On the other hand, wherever stratification by sex was implemented, no sex differences were found. Notably, none of those studies were designed or powered to specifically address the efficacy of antihypertensive treatment in women and men. A representative example is the SPRINT study, which investigated the benefit of intensive SBP reduction to levels <120 mmHg versus the conventional SBP goal <140 mmHg in elderly men and women ≥75 years of age. Women’s participation rate was only 36%, and their number of cardiovascular events was lower compared to those in the general population. The study was terminated early because of the overall benefit of intensive treatment in the male arm [122]. Similarly, negative results regarding the benefit of intensive antihypertensive treatment in women were demonstrated by two subsequent post hoc analyses [123,124]. Finally, it should be mentioned that the average age of women included in most large studies investigating the BP effect on cardiovascular events was approximately 50 years, which does not coincide with the highest prevalence of hypertension, hence excluding those postmenopausal women with the greatest cardiovascular risk [95].

### 5.2. Specific Factors Related to Antihypertensive Treatment

Several pharmacodynamic and pharmacokinetic particularities, including absorption, distribution, metabolism, and elimination of drugs, have been described in women compared to men [9,104,125]. More specifically, women have a higher gastric pH and present slower gastric emptying compared to men, which may affect the bioavailability of drugs and formulations requiring an acidic environment [126]. In addition, drug distribution is affected by a lower plasma volume, lower average tissue perfusion, and a higher percentage of body fat in women, the latter conferring a higher volume of distribution for the lipophilic substances. Finally, certain differences in cytochromes’ function have been observed, leading to significant variability in the metabolism and clearance of cardiovascular drugs [9,127].

At the same time, in daily clinical practice, certain patterns of antihypertensive drug prescription in women exist. Relative to this, a large meta-analysis of 46 population-based studies, including 164,858 women and 123,143 men aged 20–59 years, showed that hypertensive women were more likely to be treated with diuretics, while men with ACE inhibitors (ACEIs), b-blockers and calcium channel blockers (CCBs). In the same line, another meta-analysis of 43 studies, including 2,264,600 participants, showed that women were 30% more likely to be treated with diuretics and 15% less likely to be treated with ACEIs [128]. The above differences are attributed, in part, to the risk of pregnancy in younger women, therefore excluding the prescription of ACEIs and ARBs. In addition, thiazide diuretics may be preferred in women due to their decreasing effect on renal calcium, which improves bone density and reduces fracture risk in postmenopausal women. At the same time, certain drug-related side effects that appear more frequently in women compared to men constitute another factor that drives the disparities in drug prescriptions according to sex [129]. More specifically, it has been shown that women experience more frequent cough as a side effect of the treatment with ACEIs [130], suffer more frequently from lower limb edema during treatment with CCBs [131], and present more frequently electrolyte disturbances (hyponatremia, hypokalemia) with diuretics [10]. In fact, female sex has been documented as a major risk factor of thiazide-induced hyponatremia [132], and women are much more likely to be hospitalized for drug-related hyponatremia compared to men [133]. Finally, another sex-related factor that has an impact on pharmacological treatment is the higher prevalence of certain comorbidities in women, such as autoimmune diseases. Women are more susceptible to chronic and inflammatory pain and more often consume steroids and NSAIDs, which may counteract the efficacy of antihypertensive treatment due to potential side effects and a higher risk of cardiovascular complications [134,135].

### 5.3. The Role of Hormone Replacement Therapy

While estrogen depletion is a contributing mechanism to the pathogenesis of hypertension in women, on the other hand, the antihypertensive effect of hormone replacement therapy (HRT) currently remains uncertain. Overall, the available results are inconsistent since some studies have shown that HRT reduces BP [136,137], while others have shown a neutral [138,139] or even an increasing BP effect [140,141]. Similarly, the optimal formulation (estrogen alone or in combination with progestin), the most proper route of administration, and the duration of administration are all matters of concern that need to be further elucidated relative to their impact on BP and cardiovascular disease [142]. Of note, oral formulations of estrogen therapy (alone or in combination with progestins) appear to be associated with greater hypertension risk compared to other routes of administration. This association has been attributed to first-pass hepatic metabolism of oral estrogens, which has been hypothesized to result in increased RAAS activation, as evidenced by enhanced hepatic angiotensinogen production and greater downstream AngII levels [143].

Considering the above, a French prospective population-based study of 49,905 normotensive menopausal women under HRT reported that oral estrogen use, particularly in combination with a progestogen, was associated with a significantly increased risk of hypertension compared to a transdermal formulation [144]. Similarly, an 18% higher risk of hypertension was confirmed in older postmenopausal women receiving conjugated equine estrogens alone or in combination with progestins [140]. Furthermore, a more recent population-based study including 112,240 postmenopausal women who used an estrogen-only form of HRT showed that women who used the oral estrogen form had a 14% higher risk of developing hypertension compared to those using estrogen topically and 19% greater risk compared to those using vaginal creams or suppositories. At the same time, the duration of estrogen exposure and cumulative estrogen dose were positively associated with the risk of hypertension [145]. On the other hand, the Women’s Health Initiative Observational Study, including 19,986 normotensive menopausal participants, demonstrated that transdermal estradiol was associated with lower odds for newly treated hypertension compared with conjugated estrogens with or without a progestin [146].

### 5.4. Antihypertensive Treatment and BP Control in Women

Paradoxically, even though certain disparities in the antihypertensive treatment of men and women are observed, their clinical significance remains unknown. Of particular importance, current evidence broadly demonstrates comparable BP reductions with all major antihypertensive drug classes in both sexes, while no differences in the dosing regimens of antihypertensive drugs between the two sexes are proposed [11]. In addition to this, it is noteworthy that even upon starting antihypertensive therapy, women finally achieve lower rates of BP control [12,147]. This phenomenon is exacerbated with increasing age and culminates during menopause [148]. In the largest and most well-characterized cohort of postmenopausal women in the US, which included nearly 100,000 postmenopausal women of various ethnic groups aged 50–79 years, it was found that only 36.1% of hypertensive women had their BP controlled. In fact, BP control demonstrated a progressive decline with increasing age, with the lowest rate (29.3%) observed in older postmenopausal hypertensive women (70–79 years) [149]. Whether this result is solely due to physiological aging and the hormonal effects or other factors implicated, including, among others, therapeutic inertia, poor adherence, and side effects, remains to be further elucidated.

## 6. Conclusions

Hypertension is undoubtedly a major cardiovascular risk factor in women. However, there are still many controversies and unsolved questions regarding the mechanisms that lie behind its pathophysiology and impact on women. Several sex-specific factors—including substantial hormonal changes during a woman’s cycle and the plethora of other conditions that cluster with greater prevalence in females—render women more vulnerable to adverse cardiovascular events and at lower BP levels compared to age-matched men. Also, women present certain differences considering the choice of antihypertensive agent and the response to antihypertensive treatment, with subsequent poorer BP control. Despite emerging evidence, there are still many gaps regarding the proper management and treatment of female hypertensive patients, which are largely reflected in the uniform approach of hypertensive women and men in all current guidelines. More studies are imperative, aimed at a deeper understanding of the pathophysiology and therapeutic approach of hypertension in women.

## 7. Future Directions

Given the several disparities that exist considering antihypertensive treatment and cardiovascular prognosis in hypertensive women, including pre- and postmenopausal individuals, future research should focus on a deeper understanding of the sex-specific factors that drive the pathophysiology of hypertension in women compared to men. Optimal targeting of the several risk factors clustering in women is another compelling task. In addition, it is of utmost importance to increase women’s participation in large clinical trials with the aim of exploring sex-specific thresholds, targeting BP values, and improving antihypertensive regimes in order to ameliorate cardiovascular prognosis in women.

## Data Availability

No new data were created or analyzed in this study. Data sharing is not applicable to this article.

## Abbreviations

The following abbreviations are used in this manuscript:

ACEIs	angiotensin-converting enzyme inhibitors
AngII	angiotensin II
ACE	angiotensin-converting enzyme
BMI	body mass index
BP	blood pressure
CCBs	calcium channel blockers
CVD	cardiovascular disease
DM	diabetes mellitus
HFpEF	heart failure with preserved ejection fraction
HRT	hormone replacement therapy
LV	left ventricular
NO	nitric oxide
PCOS	polycystic ovary syndrome
PP	pulse pressure
PWV	pulse wave velocity
RAAS	renin angiotensin aldosterone system
SBP	systolic blood pressure
TLR	toll-like receptor
